# Stylopine: A potential natural metabolite to block vascular endothelial growth factor receptor 2 (VEGFR2) in osteosarcoma therapy

**DOI:** 10.3389/fphar.2023.1150270

**Published:** 2023-03-28

**Authors:** Naveen Kumar Velayutham, Tamilanban Thamaraikani, Shadma Wahab, Mohammad Khalid, Gobinath Ramachawolran, Shahabe Saquib Abullais, Ling Shing Wong, Mahendran Sekar, Siew Hua Gan, Angel Jemima Ebenezer, Mrinalini Ravikumar, Vetriselvan Subramaniyan, Nur Najihah Izzati Mat Rani, Yuan Seng Wu, Srikanth Jeyabalan

**Affiliations:** ^1^ Department of Pharmacology, SRM College of Pharmacy, SRM Institute of Science and Technology, Chennai, Tamil Nadu, India; ^2^ Department of Pharmacognosy, College of Pharmacy, King Khalid University, Abha, Saudi Arabia; ^3^ Department of Pharmacognosy, College of Pharmacy, Prince Sattam Bin Abdulaziz University, Al-Kharj, Saudi Arabia; ^4^ Department of Foundation, RCSI and UCD Malaysia Campus, George Town, Pulau Pinang, Malaysia; ^5^ Department of Periodontics and Community Dental Sciences, College of Dentistry, King Khalid University, Abha, Saudi Arabia; ^6^ Faculty of Health and Life Sciences, INTI International University, Nilai, Malaysia; ^7^ School of Pharmacy, Monash University Malaysia, Bandar Sunway, Subang Jaya, Selangor, Malaysia; ^8^ Center for Transdisciplinary Research, Department of Pharmacology, Saveetha Dental College, Saveetha Institute of Medical and Technical Science, Chennai, India; ^9^ Trichy Research Institute of Biotechnology Pvt Ltd, Trichy, Tamil Nadu, India; ^10^ Jeffrey Cheah School of Medicine and Health Sciences, Monash University Malaysia, Bandar Sunway, Subang Jaya, Selangor, Malaysia; ^11^ Faculty of Pharmacy and Health Sciences, Royal College of Medicine Perak, Universiti Kuala Lumpur, Ipoh, Perak, Malaysia; ^12^ Department of Biological Sciences and Centre for Virus and Vaccine Research, School of Medical and Life Sciences, Sunway University, Subang Jaya, Selangor, Malaysia; ^13^ Department of Pharmacology, Sri Ramachandra Faculty of Pharmacy, Sri Ramachandra Institute of Higher Education and Research (DU), Chennai, Tamil Nadu, India

**Keywords:** benzylisoquinoline alkaloids, stylopine, MG-63, osteosarcoma, VEGFR2

## Abstract

Vascular endothelial growth factor (VEGF) signals cell survival, cell migration, osteogenesis, cell proliferation, angiogenesis, and vascular permeability by binding to VEGF receptor 2 (VEGFR-2). Osteosarcoma is the most common primary bone cancer, majorly affects young adults. Activation of VEGFR-2 signaling is a therapeutic target for osteosarcoma. The present study aimed to evaluate the potency of stylopine in regulation of the VEGFR-2 signaling pathway and its anti-tumour effect human MG-63 osteosarcoma cells. The *in silico* study on benzylisoquinoline alkaloids was carried out for analyzing and shortlisting of compounds using a virtual screening, Lipinski’s rule, bioavailability graphical RADAR plot, pharmacokinetics, toxicity, and molecular docking studies. Among the benzylisoquinoline alkaloids, stylopine was selected and subjected to *in-vitro* studies against human MG-63 osteosarcoma cells. Various experiments such as MTT assay, EtBr/AO staining, mitochondrial membrane potential assessment, transwell migration assay, gene expression analysis by a quantitative real time polymerase chain reaction (qRT-PCR) method, SDS-PAGE followed by immunoblotting were performed to evaluate its anti-tumour effect as compared to standard axitinib. The MTT assay indicates that stylopine inhibits cell proliferation in MG-63 cells. Similarly, as confirmed by the EtBr/Ao staining method, the MMP assay indicates that stylopine induces mitochondrial membrane damage and apoptosis as compared to axitinib. Moreover, stylopine inhibits the VEGF-165 induced MG-63 cell migration by a trans-well migration assay. The immunoblotting and qRT-PCR analysis showed that stylopine inhibits the VEGF-165 induced VEGFR2 expression in MG-63 cells. It is concluded that stylopine has potential to regulate VEGFR2 and can inhibit osteosarcoma cells to offer a new drug candidate for the treatment of bone cancer in future.

## Graphical Abstract

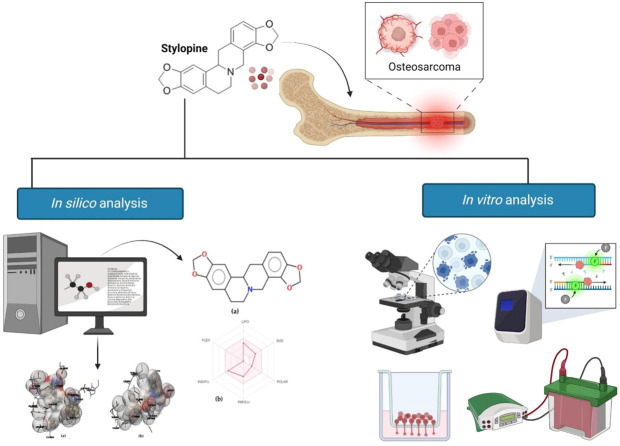

## 1 Introduction

Osteosarcoma is a tumour which is malignant in nature and affects all bones, especially the long bones in humans. It tends to mature in the later part of adulthood, showing a bimodal distribution (Moore and Luu, 2014). It is the most prevalent type of bone tumour in children and adolescents (Eaton et al., 2021). It is derived from a primitive osteoblast mesenchymal cell and is the most common primary bone malignancy. The yearly incidence of osteosarcoma in all ethnicities and genders is 4.0 (for the age groups 0–14 years) and 5.0 for the age groups 0–19 years for every million individuals (Ottaviani and Jaffe, 2009). Osteosarcoma is primarily a skeletal malignant tumour that primarily affects the long bones, where sarcoma cells create immature bone or osteoid tissue. Osteosarcoma is primarily classified clinically into two stages: localised and metastatic. Localized osteosarcoma is a type of cancer that only affects the bone and the tissues around it. Based on the viability of physically removing the tumour, it can then be divided into resectable and non-resectable stages. The metastatic stage of osteosarcoma indicates that the disease has progressed from the primary location to other organ sites, making treatment more challenging (Sadykova et al., 2020). It can be classified into subtypes based on the characteristics of the tumour and the major stromal differentiation (osteoblastic, fibroblastic, chondroblastic, small-cell, telangiectatic high-grade surface, and extraskeletal) (Sadykova et al., 2020). Clinically, the disease’s development is marked primarily by local discomfort and swelling, with occasional joint dysfunction (Zhao et al., 2021).

To date, current treatment involves immune-based targeted therapies, suppressing metastasis (Gill and Gorlick, 2021), neoadjuvant and adjuvant chemotherapy (Lugano et al., 2020), multi-agent chemotherapeutic approach with/without an aggressive surgical resection of all disease sites (Chou et al., 2008). However, these treatments are not without adverse effects, and most often produce sub-maximal effects. Hence, treatment of osteosarcoma remains to be explored, with the ideal treatment having the highest efficacy while conferring minimal side effects.

**FIGURE 1:**
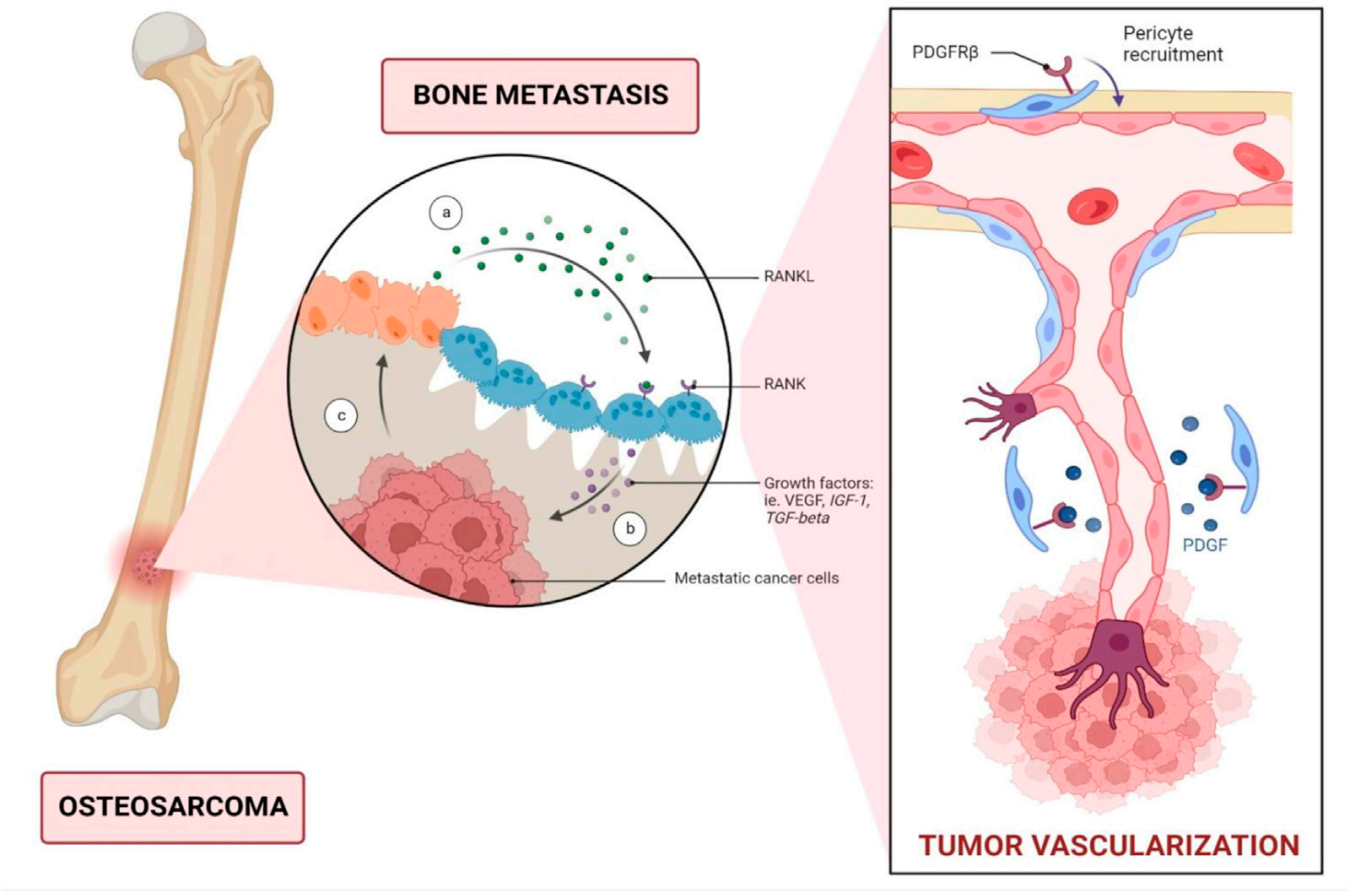
Metastatic dissemination of bone cancer through vascular endothelial growth factor (VEGF). VEGFR is a key angiogenic factor that promotes endothelial cell proliferation and consequently enhances vascular endothelial permeability. In patients with osteosarcoma, serum VEGF levels can serve as a predictive biomarker, with high VEGF expression correlated with a poor prognosis. Abbreviations: RANKL; Receptor activator of nuclear factor kappa-Β ligand; IGF-1, Insulin-like growth factor 1; TGF-beta, Transforming growth factor beta; PDGFRβ, Platelet derived growth factor receptor beta.

Angiogenesis is an essential hallmark of osteosarcoma by impacting tumour growth and its metastasis potential. Angiogenesis is often triggered by capillaries and is essential for tumour growth, maintenance, and metastasis. Several cellular pathways can cause blood vessel development in malignancies. New capillaries formation can be initiated from parental vessels (Belayneh et al., 2021). Vascular Endothelial Growth Factor (VEGF), a significant factor in angiogenesis, mainly acts on the endothelial cells (Assi et al., 2021) (Figure 1). Endothelial cells are generally quiescent, but pro-angiogenic substances such as VEGF can encourage them to sprout and initiate angiogenesis (Belayneh et al., 2021). VEGF signaling inhibition stops cell growth and initiates apoptosis in osteosarcoma. Vascular endothelial growth factor receptor 2 (VEGFR-2) and programmed death-ligand 1 (PD-L1) are expressed in approximately 64.5% and 35.5% of osteosarcoma cases respectively. They were also correlated with the PD-L1 and VEGFR2 expression in osteosarcoma when both posed a negative impact on the survival of osteosarcoma cells (Alper, 2020; Zheng et al., 2020; Assi et al., 2021). In this present study, racemic form of Stylopine was subjected for various assays. Many studies were reported using (−) Stylopine to elicit different pharmacological effect. In the current study, KEGG database was used for target selection. In VEGF (Vascular endothelial growth factor) signalling pathway, vascular endothelial growth factor receptor 2 (VEGFR2), a tyrosine kinase receptor, is stimulated by the sensitisation of ligand VEGF and causes the downstream signalling cascade mechanism intracellularly with the regulation of the various pathways including calcium, MAPK, arachidonic acid metabolism, focal adhesion turnover, actin reorganization, and PI3K-Akt, thereby leads to proliferation, migration, permeability, survival and angiogenesis of bone cells (Czarnecka et al., 2020). Thus, VEGFR2 was selected based on the consideration of its controlling of multiple effects on target cells. The racemic form of Stylopine (R,S-Stylopine), a benzyliisoquinoline alkaloid, was used for evaluating the anti-cancer effect on MG-63 cells in cellular, gene and protein levels of expression (Zheng et al., 2020). Hence, VEGFR-2 was chosen for investigation in the present study due to its high influence.

Since benzylisoquinoline alkaloids (BIA) have well-established anti-cancer activities and are available from various sources (Menendez-Perdomo and Facchini, 2018; Singh et al., 2019; Courdavault et al., 2020). Among that, stylopine has the potential for further investigation to test against human MG-63 osteosarcoma cells using *in-vitro* methods.

## 2 Materials and methods

### 2.1 *In silico* analysis of benzylisoquinoline (BIA) alkaloids

#### 2.1.1 Virtual screening

The 193 BIAs and axitinib (standard) ligands from BIAdb and PubChem databases respectively, were sketched and optimized using ChemDraw and Chem3D 16.0 Professional software. The selected human target protein (PDB ID: 4AG8) was the crystal structure of VEGFR2 kinase domain in complex with axitinib (AG-01373) (X-ray diffraction; resolution of 1.95 Å) (Yin et al., 2005). It was retrieved from RCSB Protein Data Bank (PDB) database and was optimized by deleting the hetero-atoms and water molecules. PyRx Python Prescription 0.8 tool was used for the virtual screening of test compounds and the standard against 4AG8. The Vina search space centers were 20.192, 23.739, 29.743 while the dimensions were 52.353, 51.130, 56.288 Å (Perez et al., 2012; Kostine et al., 2016; Paydas et al., 2016). Shreevatsa et al., 2021 predicted that orientin has better binding affinity towards NAD(P)H:quinone acceptor oxidoreductase-1 (NQO1) for anticancer activity using PyRx virtual screening tool (Shreevatsa et al., 2021). Vázquez-Jiménez et al., 2022 conducted ligand-based virtual screening using PyRx tool for predicting binding affinity of various benzimidazoles targeting as triosephosphate isomerase inhibitor (Vázquez-Jiménez et al., 2022).

#### 2.1.2 Lipinski’s analysis

The shortlisted benzylisoquinoline alkaloids were subjected to filters like Lipinski’s rule analysis (Molinspiration cheminformatics web-tool).

#### 2.1.3 ADMET analysis

ADMET (PreADMET web-tool) prediction and bioavailability graphical RADAR plot (Swiss ADME web-tool) were used.

#### 2.1.4 Molecular docking

An autoDock 4.2.6 software was employed for flexible docking (Lamarckian Genetic Algorithm approach with 2.5 million energy evaluations) and the binding interpretation was visualized. The modelled residues were Leu840, Val848, Lys868, Glu885, Glu917, Gly922, His1026, Leu1035, Asp1046 and Phe1047(Perez et al., 2012; Kostine et al., 2016; Paydas et al., 2016).

### 2.2 *In-vitro* analysis of stylopine on MG-63 cells

#### 2.2.1 Cell culture and treatment

MG-63 cells (Human Osteosarcoma cells) were purchased from NCCS, Pune, India. The cells were cultured in Dulbecco’s Modified Eagle Medium (DMEM), supplemented with 10% fetal bovine serum (FBS), 100 μg/ml of Streptomycin and Penicillin each. They were maintained at 37°C under 5% carbon-dioxide.

#### 2.2.2 MTT assay

MTT assay is used routinely as a benchmark for the development of novel anticancer drugs and it considered as a gold standard assay. MTT assay is regarded as the first example of a tetrazolium salt used in multi-well viability reductase-based assays for adherent mammalian. MTT assay is among the easiest cytotoxicity assays to perform. When using MTT salts, the formazan formed is water-insoluble, precipitates into cells and should be extracted with organic solvents. The end point of cell proliferation was determined by metabolic activity through MTT. This assay is well-characterized, simple to use, and referenced to this day in the literature (Tolosa et al., 2015).

The MTT reagent can pass through the cell membrane as well as the mitochondrial inner membrane of viable cells presumably due to its positive charge as well as its lipophilic structure and is reduced to formazan by metabolically active cells. ntracellular reduction of MTT can be mediated by oxidoreductase and dehydrogenase enzymes and electron donors (mainly NAD(P)H) at different stages of the glycolytic pathways to the mitochondrial electron transport chain. The location of formazan formation and its intracellular transportation has remained controversial. While the role of mitochondria in MTT reduction has been a justification for the common application of the assay to measure mitochondrial activity, biochemical and microscopic studies have located formazan in various intracellular organelles. Intracellular formazan granules have been observed in the nucleus, microsomes, endoplasmic reticulum, plasma membranes, and cytosolic lipid droplets. These observations suggest that the MTT assay is more than a mere representation of mitochondrial activity (Ghasemi et al., 2021).

Bahuguna et al. (2017) reported that MTT assay for cytotoxicity evaluation has many advantages in especially its effectiveness and simplicity, which make it more suitable to assess the anti-cancer potential of any test samples at preliminary levels (Bahuguna et al., 2017). Zhang et al. (2021) demonstrated the cytotoxic effect of dieckol in human osteosarcoma MG-63 cells using MTT assay (Zhang et al., 2021). Liu et al. (2015) revealed that the combination of ZD6474 and celecoxib had a stronger antiproliferative effect in human osteosarcoma cells (MG-63) through MTT assay (Liu et al., 2015).

To evaluate the cytotoxicity, MG-63 cells (1×10^5^ cells/ml) were treated with 0.5, 1, 5, 10 and 50 µM of compounds (test and standard) in triplicates. After 24 h of incubation, each well was treated with 20 µl of MTT (5 mg/ml) and was incubated until purple-colored precipitates were visible (2–4 h). A Thermo Fisher Scientific microplate reader was used to measure the absorbance at 570 nm for the IC50 values, which were used for further assays (Dahlin et al., 1970; Renema et al., 2016; Rosario et al., 2017).

#### 2.2.3 EtBr/AO staining

To assess the mechanism of cell death, approximately 5 × 10^5^ cells/ml were treated with both compounds (test and standard). After incubation, 50 µl of acridine orange (1 mg/ml) and ethidium bromide were added. The solution was evaluated within an hour using a fluorescence filter (Cornélio et al., 2011).

#### 2.2.4 JC-10 staining

Approximately 5,000–20,000 cells/well were treated with the two compounds (test and standard). The cells were incubated with 100 μl/well JC-10 dye loading solution and an assay buffer B. The solution was kept protected from any light. Fluorescence was measured at 490/525 nm and 540/590 nm (Ashraf et al., 2020).

#### 2.2.5 Transwell migration assay

The effect of the compounds on cell invasion was assessed using Transwell chambers (8 µm pore size, Corning, United States of America), from the lower chamber. The cells were placed on the microporous membrane. After incubation, the non-invasive cells on the upper surface were removed, while the invasive cells on the lower chamber were fixed (75% ethanol) and stained (0.5% crystal violet). The results were presented as images of the invading cells (Dahlin et al., 1970; Renema et al., 2016; Rosario et al., 2017).

#### 2.2.6 qRT-PCR method

Total RNA isolation was performed through TRIZOL method according to the manufacturer’s instruction. The samples were then centrifuged *via* a diethylpyrocarbonate DEPC-treated centrifuge tube at 5,000 rpm for 10 min to obtain the cell pellet. To the cell pellet (1 × 10^7^ cells), 700 µl of TRIZOL was added to allow cell lysis. The lysate was collected into 1.5 ml tubes and was vigorously pipetted out. Then, 300 µl of chloroform was added. The solution was vigorously mixed for 5 min at room temperature.

The aqueous layer was separated by centrifugation at 12,000 rpm for 20 min at 4°C. The aqueous layer was then collected into a fresh 1.5 ml tube. RNA was later precipitated by the addition of isopropanol (700 µl). The precipitated RNA was pelleted by centrifugation at 12,000 rpm for 20 min at 4°C. The pellet was then washed with 70% ethanol. Finally, the air-dried RNA pellet was dissolved into double distilled autoclaved water (30 µl) and stored at −80°C until subsequent use. The quantity and quality of the isolated RNA was estimated by Labman UV Vis Spectrometer on a 1.5% agarose gel.

DNase was added, in case of DNA contamination with the RNA preparation. The reaction volume was set to 20 µl with 1U of DNase. The solution was incubated at 37°C for 30–45 min. Subsequently, 20 µM of 2 µl ethylene glycol tetra-acetic acid (EGTA) was added. The solution was incubated at 66 °C for 10 min. Then, sodium acetate (1/10 V) and absolute ethanol (2V) were added, and the solution was incubated at −20°C for 60 min. The step was followed by a centrifugation step at 12,000 rpm for 20 min at 4°C, where the supernatant was discarded. Finally, the pellet was washed with 500 µl of 75% ethanol. The air-dried pellets were dissolved in 20 µl of double autoclaved Milli-Q grade water and was stored until next use.

Total RNA was converted to cDNA by using a reaction mixture containing a reverse transcriptase (MMLV). The cDNA synthesis was conducted at 25°C (10 min) followed by 37°C (120 min). Denaturation of cDNA and RNA hybrid and reverse transcriptase inactivation were conducted at 85°C for 2 min. The yielded cDNA was then used as a template for detecting metastasis. Then, a qRT-PCR was conducted by using a Power Syber Green kit (Applied Biosystems, CA, United States) in ABI StepOne Plus (Applied Biosystems, CA, United States). The expression of the selected genes (Primer sequence) was assessed by qRT-PCR using the relative quantification (2^-^∆∆CT^) method (Table 1). Expression was normalized using the endogenous control (GAPDH) while control cells were used as the calibrator.

**TABLE 1:** Gene primer sequences used for qRT-PCR analysis.

Target gene	Primer sequence used
Human VEGFR2	3′-CTG​GGA​ATC​CCC​CTC​CAC​AG-5′
	5′- GCG​GAT​AGT​GAG​GTT​CCG​GT-3′
Human GAPDH	3′-TGA​CTT​CAA​CAG​CGA​CAC​CCA-5′
	5′-CAC​CCT​GTT​GCT​GTA​GCC​AAA-3′

An initial melting temperature of 94°C for 15 min, followed by 40 cycles of 95°C for 10 s was used. The annealing temperature was set at 52°C for 15 s while extension was set for 72°C (20 s). The real time data was captured at the end of each extension stage. The step was followed by the melting curve analysis as per the default temperature profile of the Thermal cycler (Dahlin et al., 1970; Renema et al., 2016; Rosario et al., 2017).

#### 2.2.7 Western blotting

The cells were washed twice with an ice-cold phosphate buffer saline (PBS) by centrifugation at 2000 rpm for 5 min at 4°C. Briefly, PBS was aspirated and then ice-cold radioimmunoprecipitation assay buffer (RIPA) buffer was added (1 × 10^6^ cells/100 µl). The solution was subjected to a constant agitation for 30 min at 4°C. Repeated pipetting was conducted to shear the DNA to reduce sample viscosity. The solution was spinned at 10,000 rpm for 10 min at 4°C, before being pre-cooled, and centrifuged. The supernatant was collected and was stored at −20°C until further use. The protein concentration was calculated by using a Bradford assay. Protein sample (50–100 µg) was taken and SDS sample loading dye was added (1:2). The sample was boiled further at 95°C for 5 min. Equal amounts of protein was loaded into the wells. The gel was ran at 50 V for 5 min. Finally, the voltage was increased to 100 V and the run was stopped after an hour.

The gel was placed in a 1X transfer buffer for 10–15 min. The transfer sandwich was assembled to ensure that there were no air bubbles trapped in the sandwich. The blot was on the cathode while the gel was on the anode. The cassette was placed in the transfer tank that was set in an ice block in the tank before being further transferred at 100 V for 120 min. The blot was rinsed in water and was stained with ponceau-S solution and the transfer quality was checked. The ponceau-S stain was rinsed with three washes of PBS with tween (PBST). The solution was blocked with 5% skim milk in PBST at room temperature for 1 h. Subsequently, the solution was subjected to an overnight incubation in a primary antibody solution against the target protein at 4°C. The blot was rinsed further 3–5 times for 5 min with PBST. Finally, the solution was incubated in a horseradish peroxidase (HRP)-conjugated secondary antibody solution for 1 h at room temperature. The blot was rinsed (3–5 times) with PBST.

Subsequently, 3, 3′-diaminobenzidine (DAB) and hydrogen peroxide solution (1:1) was dissolved in PBS. The mixture was slowly added to the blot. The stained protein band expression was captured as an image using a digital camera (Dahlin et al., 1970; Renema et al., 2016; Rosario et al., 2017).

## 3 Results and discussion

### 3.1 *In-silico* analysis of benzylisoquinoline (BIA) alkaloids

#### 3.1.1 Virtual screening

Virtual screening of benzylisoquinoline alkaloids against human VEGRF2 kinase domain (PDB ID: 4AG8) using a PyRx tool yielded the binding affinity of all the 193 compounds and that of the standard axitinib. The binding energy (kcal/mol) of 1-methoxyberberium, acetylcorynoline, actinodaphine, adiantifoline, aknadicine, alpinine, alamarine, alangicine, alangimarine, allocryptopine, alpha-erythroidine, amurensine, ancistrocladine, angoline, ankorine, anolobine, anonaine, armepavine, asimilobine-2-O-glucoside, aromoline, atheroline, atropine, beberine, berbamine, backebergine, berberal, berberine, berberine chloride, beta-erythroidine, bicuculline, bianfugedine, bisnordihydrotoxiferine, boldine, bracteoline, bulbocapnine, caffeine, canadine, capaurine, caryachine, C-curarine, cephaelin, cephalotaxine, cepharanthine, chelirubine, coclaurine, codeine, colchicine, columbamine, coptisine, corpaine, corydaline, crebanine, cularicine, cularidine, cularimine, cularine, cycleanine, daphnandrine, daphnoline, dauricine, daurisoline, dehydrocorydalin, dehyrocrebanine, dehydrostephanine, demecolcine, dicentrine, dihydrosanguinarine, DL-laudanine, drotaverin, emetamine, emetine, erysonine, erysotrine, erythratidine, eschscholtzidine, fagaridine, fagaronine, fetidine, fugapavine, fumaricine, gigantine, glaucine, glaziovine, gyrocarpine, hasubanonine, heliamine, hernandezine, higenamine, homochelidonine, homotrilobin, hydrastine, ipecoside, isococculidine, isocorydine, isocorypalmine, isoteolin, isothebaine, isotrilobine-N-2-oxide, jatrorrhizine, laudanosine, laurifine, laurifoline chloride, laurolitsine, laurotetanine, liriodenine, longifolonine, lophocerine, lophophorine, lysicamine, macarpine, macoline, magnoflorine, menisperine, metocurine, morphine, nandazurine, nantenine, narcein, narcotoline, neferine, neopine, nitidine, n-methylnandigerine, nordicentrine, norlaureline, norstephalagine, nuciferine, obaberine, oblongine, ochotensine, ocoteine, oliveroline, O-methylbulbocapnine, opium, oxoaporphine, oxoglaucine, oxophoebine, oxopurpureine, oxyacanthine, palmatine, papaveraldine, papaverine, perfumine, pellotine, pessoine, phaeanthrine, phellodendrine, pilocercine, polycarpine, predicentrine, pronuciferine, protopine, psychotrine, pukateine, puterine, reticuline, rhoeadine (R)-N-methylcoclaurine, rodiasine, roemerine, rugosinone, salsoline, salutaridine, sanguinarine, scopolamine, sinomenine, spinosine, (s)-scoulerine, stepharine, stepholidine, stylopine, tetrahydrocolumbamine, tetrahydropalmatine, tetrandrine, thalcimine, thalicarpine, thalicberine, thalicminine, thalicsimidine, thalidasine, thalifaberidine, thaliporphine, thalmidine, thalmine, thebaine, tiliacorine, toxiferine, trilobine, tubocurarine, ukrain, xylopine, xylopinine, zijinlongine, and standard axitinib were −7.7, −8.2, −8.8, −8.7, −7.5, −8.2, −8.9, −8.6, −8.5, −8.1, −7, −7.5, −7.4, −7.7, −7.5, −7.9, −7.9, −7, −8, −8.5, −8.3, −7.5, −9.4, −8.4, −6.3, −8.7, −8.3, −7.8, −6.8, −9, −8.5, −9.9, −7.9, −7.9, −8.1, −5.6, −9.5, −7.9, −7.5, −9.6, −8.2, −7.7, −9.9, −9.7, −7.7, −7.7, −7.1, −8, −9.5, −8.6, −8.2, −8.3, −8.6, −8.3, −8, −8.2, −8.5, −9, −9, −9.8, −9.2, −7.6, −8.1, −7.9, −6.8, −8.8, −9.4, −7.6, −8.6, −8.4, −8.2, −7.1, −7.3, −7.3, −7.5, −9, −8.7, −9.4, −8, −7.9, −6.4, −7.8, −7.8, −8.8, −6.9, −6.5, −8.7, −9.4, −9, −6.9, −8.1, −7.3, −9.2, −7.9, −8.1, −7.9, −7.9, −8.8, −8.8, −7.8, −9.2, −7.8, −8.2, −8.3, −8.3, −7.9, −6.5, −7.1, −7.9, −9.1, −7.6, −7.6, −7, −8.7, −7.6, −8.7, −8.8, −8.3, −7.6, −7.8, −7.9, −9.1, −7.7, −8, −7.9, −8.4, −7.9, −7.6, −7.4, −7.7, −8.8, −8.2, −8.8, −7.2, −8.3, −8.3, −7.7, −8.3, −9.3, −8, −7.5, −7.3, −8, −6.3, −9.4, −8.7, −8.5, −6.5, −8.2, −8.2, −7.9, −8, −8.9, −7.9, −7.9, −8.8, −8.1, −7.1, −8.8, −7.9, −7.5, −6.5, −8.8, −9.5, −7.3, −7.6, −9.4, −8.1, −7.5, −7.7, −10.9, −7.8, −8, −9.4, −8, −8.6, −8.5, −8.2, −8.3, −8.5, −8.5, −8.8, −8, −8.4, −8.9, −9.6, −9.6, −9, −9.3, −8.3, −7.9, −7.5, −7.7, and −9.7 kcal/mol, respectively.

From the 193 compounds, three alkaloidal compounds 44/Chelirubine (−9.7 kcal/mol), 60/Dauricine (−9.8 kcal/mol), and 171/stylopine (−10.9 kcal/mol) were shortlisted for comparison with the binding affinity of standard axitinib (−9.7 kcal/mol).

#### 3.1.2 Lipinski’s analysis of chelirubine, dauricine, and stylopine

Lipinski’s rule analysis of the selected three compounds through Molinspiration tool yielded miLogP reading of 0.78, TPSA of 50.05, natoms of 27, molecular weight (MW) of 362.36, nON of 6, nOHNH of 0, nviolations of 0, nrotb of 1, and volume of 305.06 for compound chelirubine; miLogP of 5.98, topological polar surface area (TPSA) of 72.87, natoms of 46, MW of 624.78, nON of 8, nOHNH of 1, nviolations of 2, nrotb of 10, and volume of 588.93 for compound dauricine; miLogP of 3.04, TPSA of 40.17, natoms of 24, MW of 323.35, nON of 5, nOHNH of 0, nviolations of 0, nrotb of 0, and volume of 278.45 for stylopine.

#### 3.1.3 Absorption, distribution, metabolism, excretion (ADMET) analysis of chelirubine, dauricine, and stylopine

PreADMET tool was used to predict the toxicity and pharmacokinetic properties of the three selected compounds. The results are indicated within brackets in the order of chelirubine, dauricine, stylopine next to each parameter evaluated. Under toxicity prediction, parameters like algae at (0.0832,285, 0.00148,914, 0.0391,758), Ames_test (mutagen, non-mutagen, mutagen), Carcino_Mouse (negative, negative, negative), Carcino_Rat (positive, positive, negative), daphnia_at (0.108,198, 0.00377,309, 0.0954,573), hERG_inibition (medium_risk, medium_risk, low_risk), medaka_at (0.0259,675, 4.68229e-005, 0.0148,898), minnow_at (0.0523,462, 0.000175,359, 0.0206,951), TA100_10RLI (negative, negative, negative), TA100_NA (negative, negative, negative), TA1535_10RLI (negative, negative, negative), and TA1535_NA (negative, negative, negative) were evaluated.

Under pharmacokinetic prediction, parameters like BBB (0.55219, 0.192,428, 0.0326,085), Buffer_solubility_mg/L (0.230,166, 0.197,671, 2.16526), Caco2 (55.7346, 50.6098, 54.3514), CYP_2C19_inhibition (Inhibitor, Non, Non), CYP_2C9_inhibition (Inhibitor, Inhibitor, Inhibitor), CYP_2D6_inhibition (Inhibitor, Non, Inhibitor), CYP_2D6_substrate (Substrate, Substrate, Substrate), CYP_3A4_inhibition (Inhibitor, Inhibitor, Inhibitor), CYP_3A4_substrate (Substrate, Substrate, Substrate), HIA (97.705,735, 97.508,797, 97.787,081), MDCK (17.182, 0.0599,765, 53.1927), Pgp_inhibition (Inhibitor, Inhibitor, Non), Plasma_Protein_Binding (63.964,295, 75.847,807, 79.604,608), Pure_water_solubility_mg_L (0.0234,203, 1.99174, 10.2783), Skin_Permeability (−4.57469, −2.72815, −4.45675), SKlogD_value (0.897,530, 2.673,310, 1.557,810), SKlogP_value (0.897,530, 5.802,230, 3.122,270), SKlogS_buffer (−6.197,100, −6.501,180, −5.174,160), and SKlogS_pure (−7.189,550, −5.497,890, −4.497,750) were evaluated.

**FIGURE 2:**
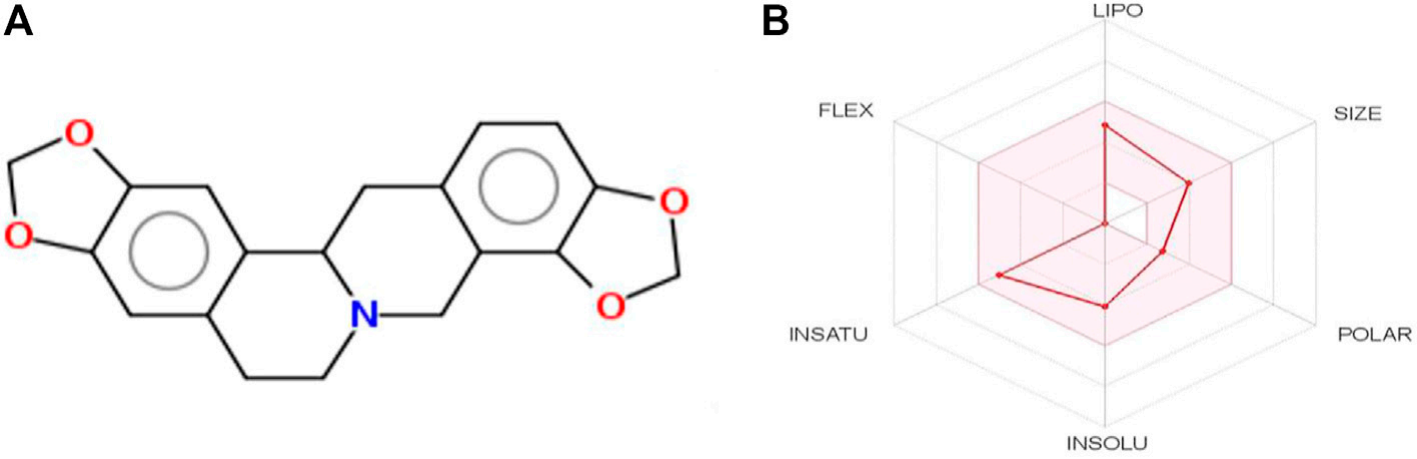
**(A)** Two-dimensional (2D) structure of stylopine; **(B)** Bioavailability graphical RADAR plot of stylopine using SwissADME web-based tool.

Among the three tested compounds, stylopine was predicted as a potential compound based on various parameters such as the binding affinity, drug likeness analysis, pharmacokinetics, toxicity and binding interactions, that have been generated for a set of benzylisoquinoline alkaloids using various softwares/tool such as PyRx, Molinspiration Cheminformatics, Swiss ADME, and PreADMET. The selected test compound stylopine was subjected to Swiss ADME web tool to generate its bioavailability graphical RADAR plot (Figure 2). Hence, stylopine was chosen for further *in silico* evaluation with human VEGFR2 kinase.

#### 3.1.4 Molecular docking of stylopine with human VEGFR2 kinase

**FIGURE 3:**
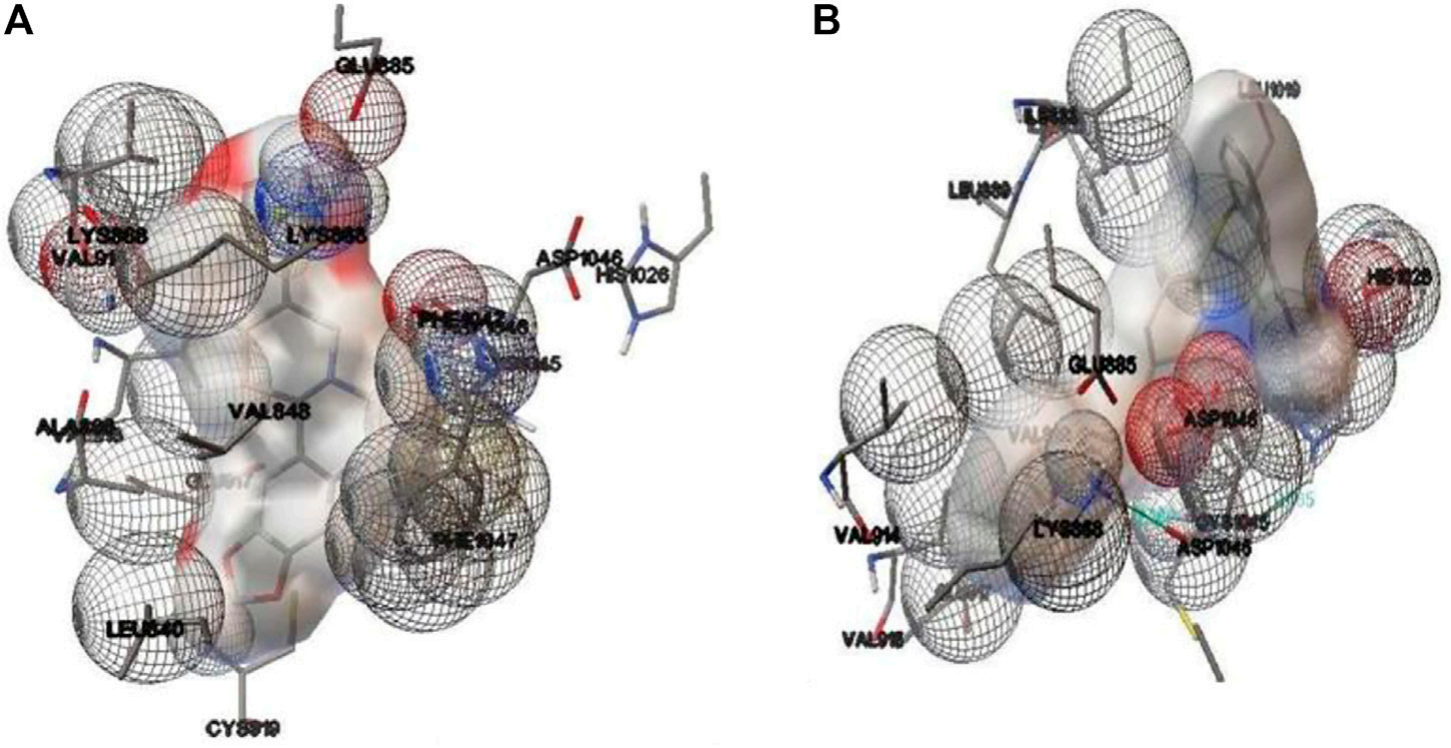
Binding interactions involved in the docked complex of **(A)** stylopine (test compound) **(B)** axitinib (standard compound) with human VEGFR2 kinase domain (PDB ID: 4AG8) using an AutoDock 4.2.6 software.

Molecular docking results for the docked complex of the selected test compound stylopine and standard axitinib with human VEGFR2 kinase domain were evaluated using parameters like the binding_energy (−10.1, −9.28 kcal/mol), ligand_efficiency (−0.42, −0.33), inhib_constant (39.52 nM, 156.94 nM), intermol_energy (−10.10, −10.77), vdw_hb_desolv_energy (−4.68, −3.42), electrostatic_energy (0.20, −0.05), moving_ligand_fixed_receptor (−5.62, −7.30), moving_ligand_moving_receptor (−4.48, −3.47), total_internal (−18.32, −18.07), ligand_internal (0.00, 0.00), torsional_energy (0.00, 1.49), unbound_energy (−18.32, −18.07), cIRMS (0.00, 0.00), refRMS (51.14, 45.96), rseed1 (None, None), and rseed2 (None, None) respectively using AutoDock 4.2.6 software (Figure 3).

The findings indicated that stylopine (−10.1 kcal/mol) has a better binding affinity towards VEGFR2 kinase domain (PDB ID: 4AG8) when compared to the standard compound axitinib (−9.28 kcal/mol). Hence, it was hypothesized that stylopine (39.52 nM) is potent against VEGF receptor two when compared to the standard axitinib (156.94 nM). Interestingly, two and one *π*-cation interactions formed in the docked complexes stylopine-Lysine 868 residue of VEGFR2 and Axitinib-Lysine 868 residue of VEGFR2 respectively. There was no hydrogen bond formation. There were also no *π*-π stacking interactions formed in the docked complexes. Thus, the *in silico* molecular docking study predicts that test ligand stylopine may be a potential compound as compared to the standard axitinib against the human VEGFR2 tyrosine domain (Rahman et al., 2021; Chabukswar et al., 2022). Hence, stylopine was further investigated on MG-63 cells using *in-vitro* methods.

### 3.2 *In-vitro* analysis of stylopine on MG-63 cells

**FIGURE 4:**
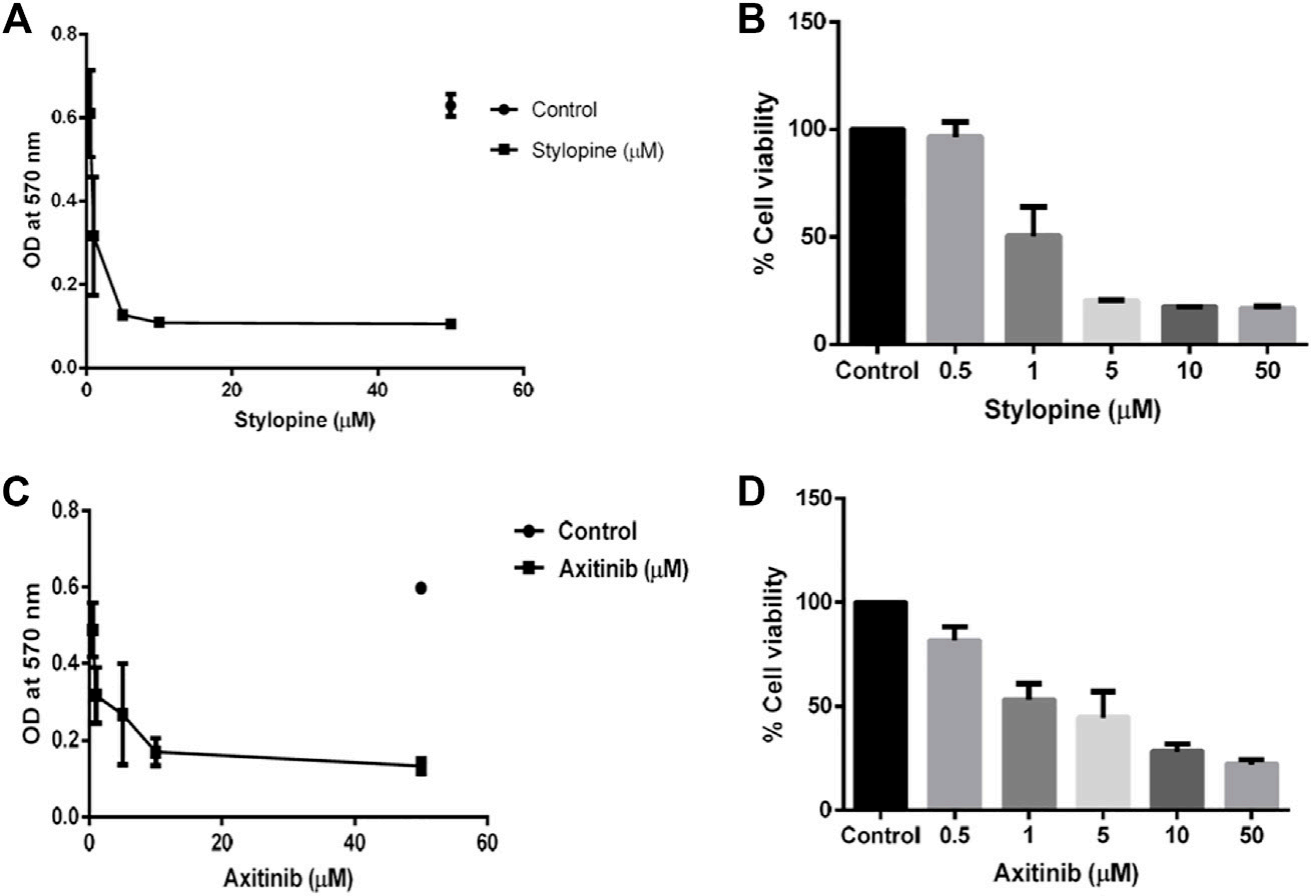
MTT assay findings using MG-63 cells. **(A)** Optical density (OD) value of stylopine-treated cells; **(B)** the percentage cell viability of stylopine-treated cells; **(C)** OD value of standard axitinib-treated cells **(D)** the percentage cell viability of axitinib-treated cells.

**FIGURE 5:**
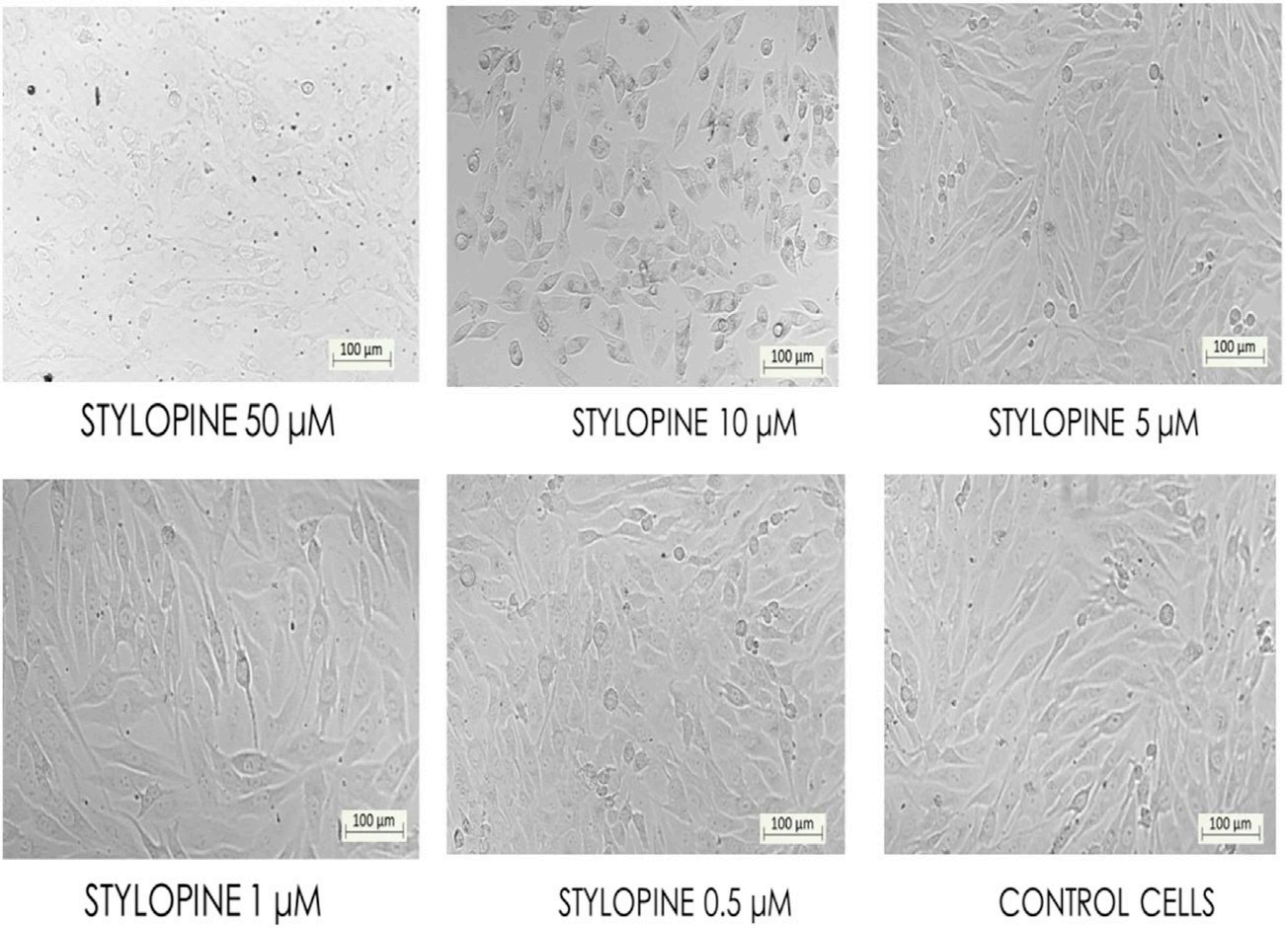
Bio-imaging of control and different concentrations of stylopine-treated MG-63 cells by MTT assay (Magnification - ×10).

**FIGURE 6:**
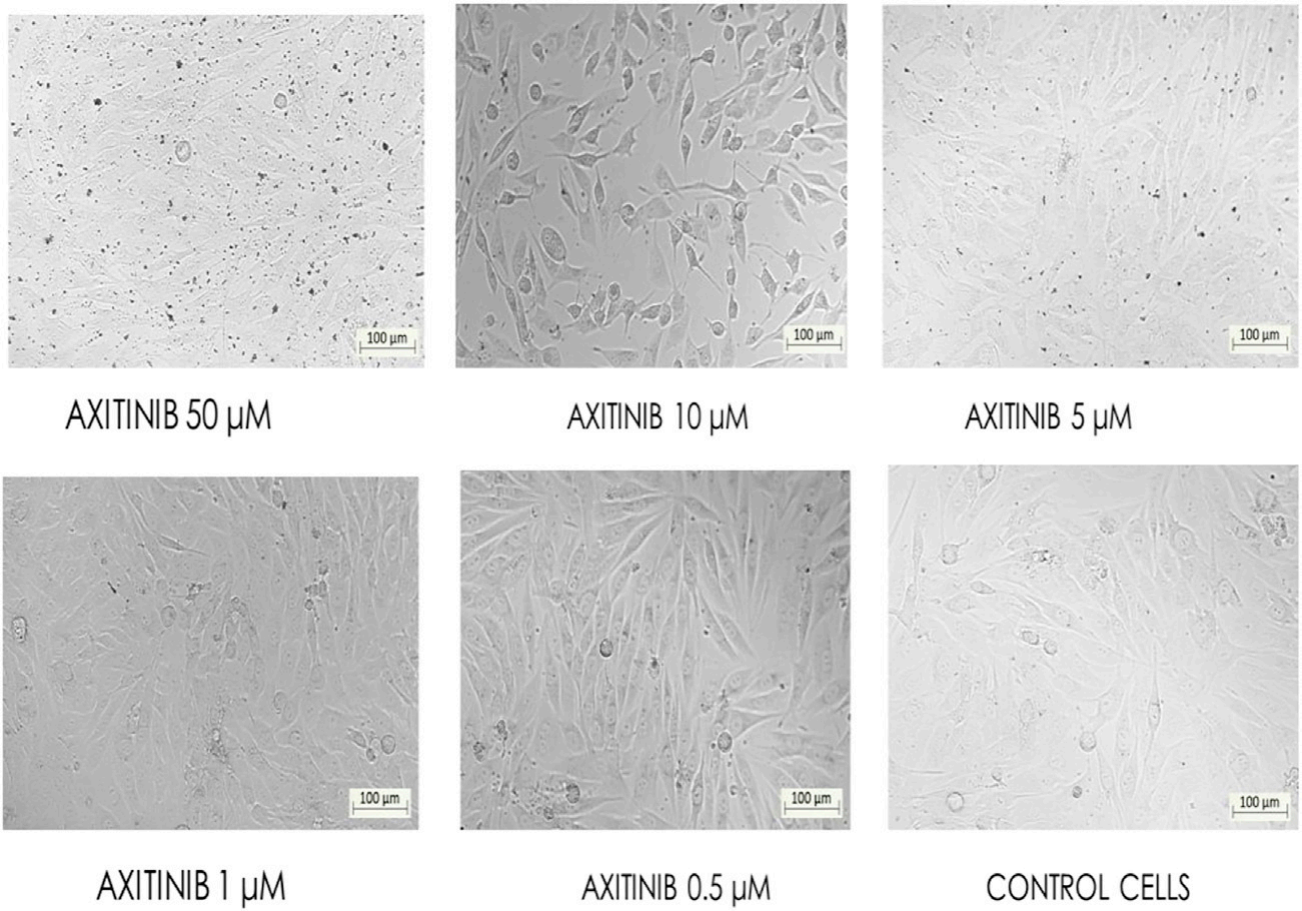
Bio-imaging of control and different concentrations of axitinib-treated MG-63 cells as confirmed by the MTT assay (Magnification - ×10).

*In-vitro* cell line studies were performed to evaluate the effect of stylopine on human Osteosarcoma MG-63 cells when compared with the standard axitinib. MTT assay results indicated that the IC_50_ value of the test compound stylopine and standard axitinib were 0.987 µM and 2.107 µM respectively (Figures 4–6).

**FIGURE 7:**
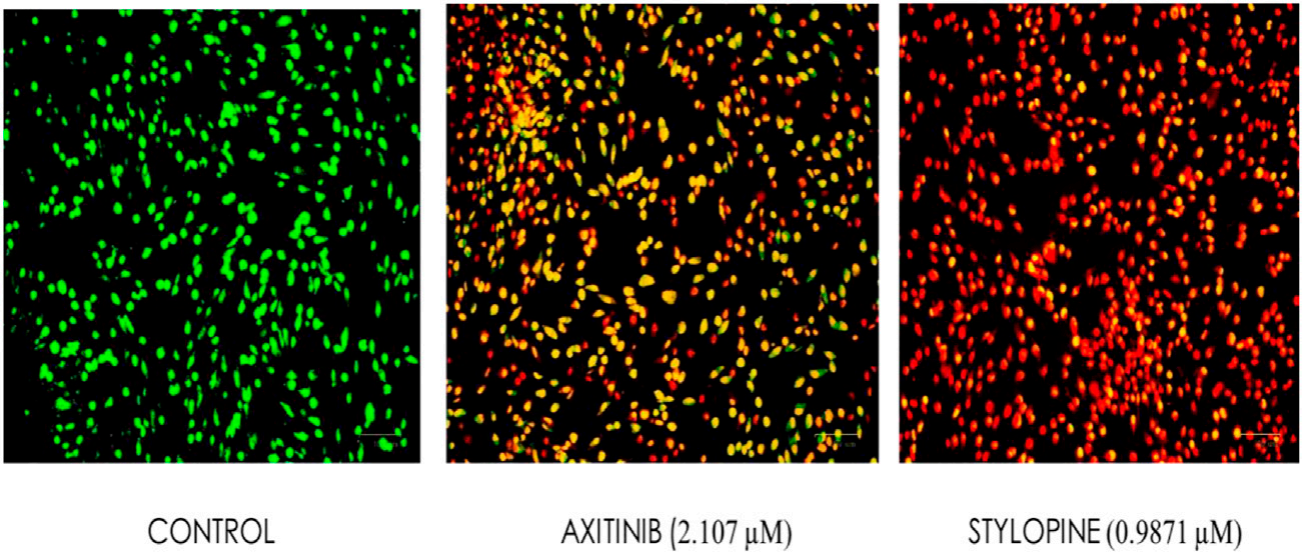
Fluorescence-based cell death assessment of human osteosarcoma MG-63 cyto-imaging by EtBr/AO staining. Green, red, yellowish green, yellowish orange, and orange red-colored cells indicate live, dead, early apoptotic, late apoptotic, and necrotic cells respectively.

**FIGURE 8:**
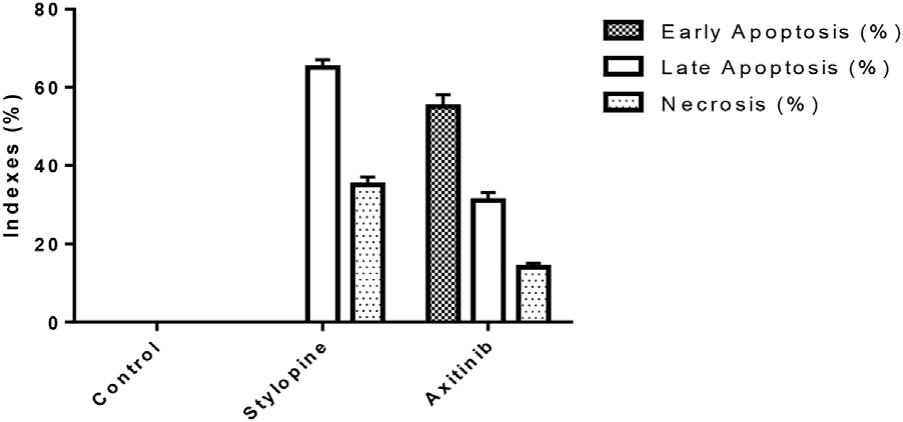
Indexes of early, late apoptosis and necrosis of control and treated MG-63 cells by EtBr/AO staining method; ****p < 0.0001, statistically significant data by a two-way ANOVA.

Ethidium bromide and acridine orange staining procedure confirmed that treatment with 0.9871 µM stylopine induced the MG63 cells to undergo apoptosis, while a higher concentration (2.107 µM) was required for the standard axitinib (Figures 7, 8).

**FIGURE 9:**
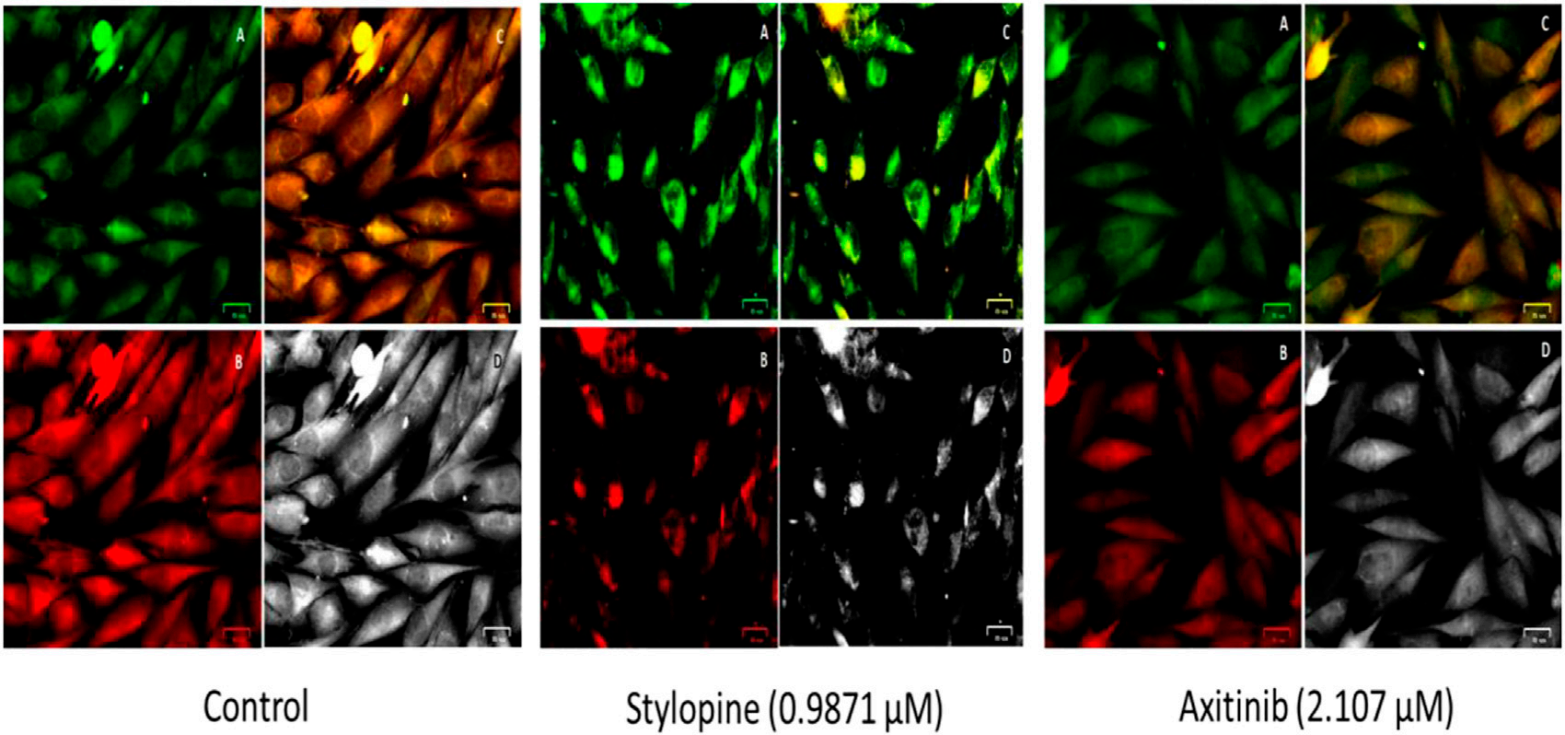
Fluorescence based bio-imaging assessment of disruption in the mitochondrial membrane of MG-63 cells by JC-10 staining method. Green, red, merged green and red filtered fluorescence images are designated as A, B and C, respectively, whereas D represents the bright field image.

The findings from the JC-10 dye study showed that treatment with 0.9871 µM stylopine disrupted the mitochondria when compared to the 2.107 µM of standard axitinib-treated MG-63 cells (Figure 9).

**FIGURE 10:**
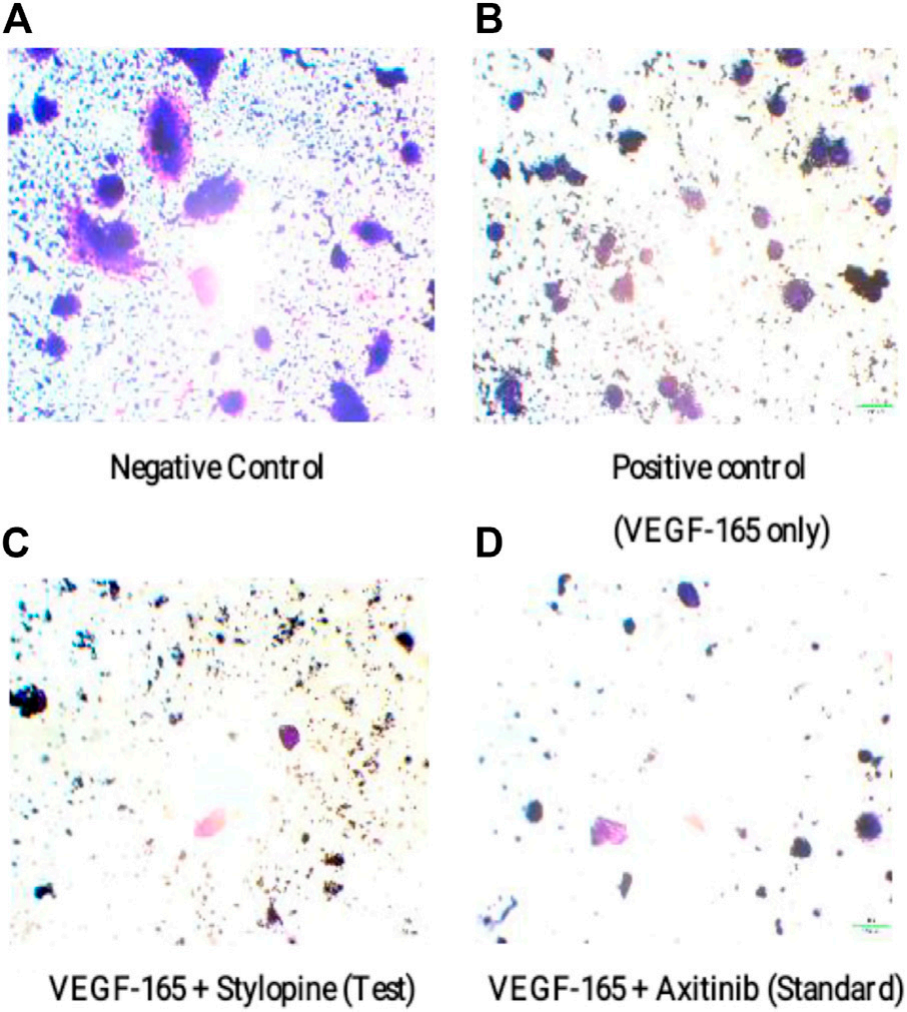
Representation of migrated MG-63 cells using an inverted microscope based on transwell migration assay. **(A)** negative control, **(B)** cells treated with VEGF-165 only, **(C)** cells treated with VEGF-165 with stylopine, and **(D)** cells treated with axitinib.

Furthermore, the microscopic observations showed that stylopine decreased the number of migrated MG-63 cells on both test and standard wells as compared to the positive and negative controls. Based on the transwell invasion assay, we conclude that stylopine can potentially reduce chemotaxis of human MG-63 cells (Figure 10).

**FIGURE 11:**
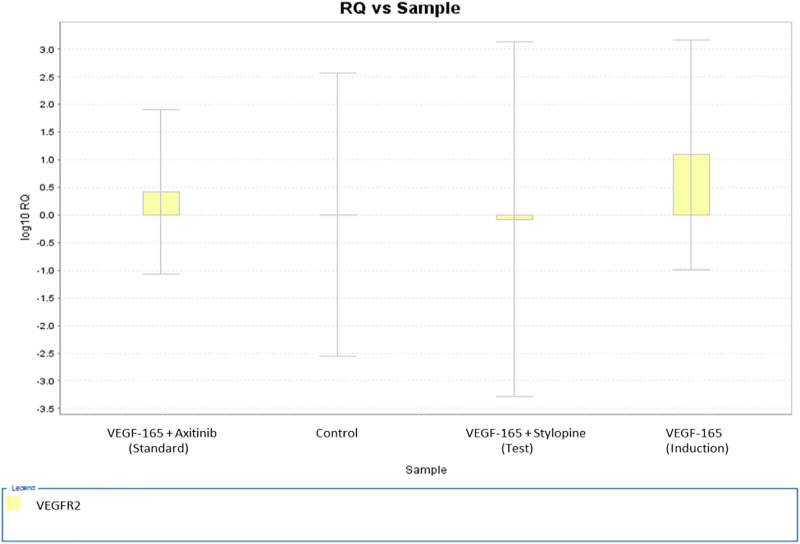
Gene expression plot for the target VEGFR2 representing a fold variation (log 10 relative quantification) vs sample on MG-63 cells. The values of fold variations are 0 (control), 1.09 (induction), 0.4 (standard), and −0.07 (test).

Gene expression analysis of VEGFR2 performed *via* a quantitative real time polymerase chain reaction (qRT-PCR) technique (double delta threshold cycle method) showed that stylopine induced the downregulation of VEGFR2 gene expression, whereas treatment with standard axitinib reduced the gene expression on treated human osteosarcoma MG-63 cells. Treatment with VEGF-165 led to the upregulation of VEGFR2 gene when compared with control (Figure 11).

**FIGURE 12:**
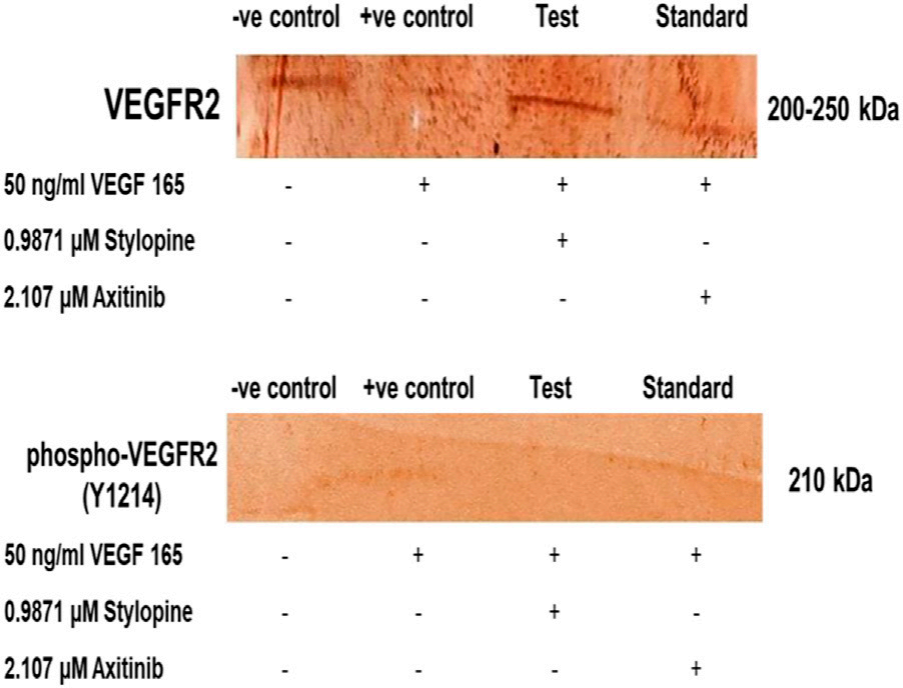
Target protein expression on MG-63 cells using SDS-PAGE followed by immune-blotting and DAB staining.

The MG 63 cells were activated with VEGF-165 ligand followed by treatment with the IC_50_ concentrations of stylopine and axitinib. Their responses to VEGFR2 (Y1214) were evaluated using a sodium dodecyl-sulfate polyacrylamide gel electrophoresis (SDS-PAGE), followed by an immune-blotting technique. The results showed that VEGF-165 induced phosphorylation of VEGFR2 protein and the expression of total VEGFR2 protein in MG-63 cells. Treatment with stylopine (0.9871 µM) significantly 1) inhibited the phosphorylation of VEGFR2 and 2) reduced the expression of total VEGFR2 as compared to the standard axitinib drug (2.107 µM). Taken together, these results showed that stylopine can inhibit VEGF-165 induced VEGFR2 expression in MG-63 cells (Figure 12). Hence, stylopine causes an inactivation of downstream signaling molecules in VEGFR2 signaling pathway which contributes to the pathogenesis of osteosarcoma (Wang et al., 2019; Kumar et al., 2021).

Wang et al. (2019) revealed that anlotinib acts as VEGFR2 antagonist on MG-63 cells thereby inhibits the growth of osteosarcoma, angiogenesis and metastasis (Wang et al., 2019). Lee et al. (2018) betrayed that suppression of bone tumor growth by cabozantinib collectively inhibited VEGFR2 and c-Met in osteoblasts with the association of reduced tumor-induced osteolysis (Lee et al., 2018). Liu et al. (2017) reported that apatinib as a highly selective VEGFR2 antagonist which, has a promising antitumoral effect by the growth inhibition of osteosarcoma cells *in vitro* and *in vivo* through apoptosis, cell cycle arrest, and autophagy (Liu et al., 2017). Zhang et al. (2021) evaluated that the effective inhibition of dieckol in the PI3K/AKT/mTOR signaling on the MG-63 cells (Zhang et al., 2021).

## 4 Conclusion

In the present study, computer-aided drug design approach involved in a successful screening process of selected chemical library containing 193 benzylisoquinoline alkaloids. The phytoalkaloid stylopine was predicted as a better compound with the consideration of parameters such as binding affinity, drug likeness analysis, pharmacokinetics, toxicity and binding interactions, which were generated for set of benzylisoquinoline alkaloids using various softwares/tool such as PyRx, Molinspiration Cheminformatics, Swiss ADME, PreADMET and AutoDock 4.2.6. Further, the *in vitro* characterization of stylopine in comparison with standard Axitinib were carried out in MG-63 cells. The MTT results showed that the stylopine could inhibit the proliferation of MG-63 cells than the standard Axitinib. Further, the effect of test compound stylopine on the expression of VEGFR2 gene and protein expression was evaluated by RT-PCR and Immunoblotting analysis in MG-63 cells. The results showed that the activation with VEGF-165 ligand induced the expression of VEGFR2 gene and induced the phosphorylation of VEGFR2 protein, Whereas, stylopine inhibited VEGF-165 ligand induced the expression of VEGFR2 protein and gene expression. The cellular mechanism of stylopine was studied using MMP assay, ETBr/AO staining and transwell migration assay in comparison with standard Axitinib. The results showed that the stylopine caused the mitochondial membrane damage (MMP assay) and leads to apoptosis (EtBr/AO) and inhibited the cell migration (Transwell migration) in MG-63 cells. In summary, our findings indicated that stylopine has an anticancer activity by inhibiting the expression of VEGFR2 gene and inducing apoptosis in MG-63 cells. In future, the preclinical and clinical studies of stylopine need to be conducted to develop stylopine as a potential drug for bone cancer treatment.

## 5 Future prospects

**FIGURE 13:**
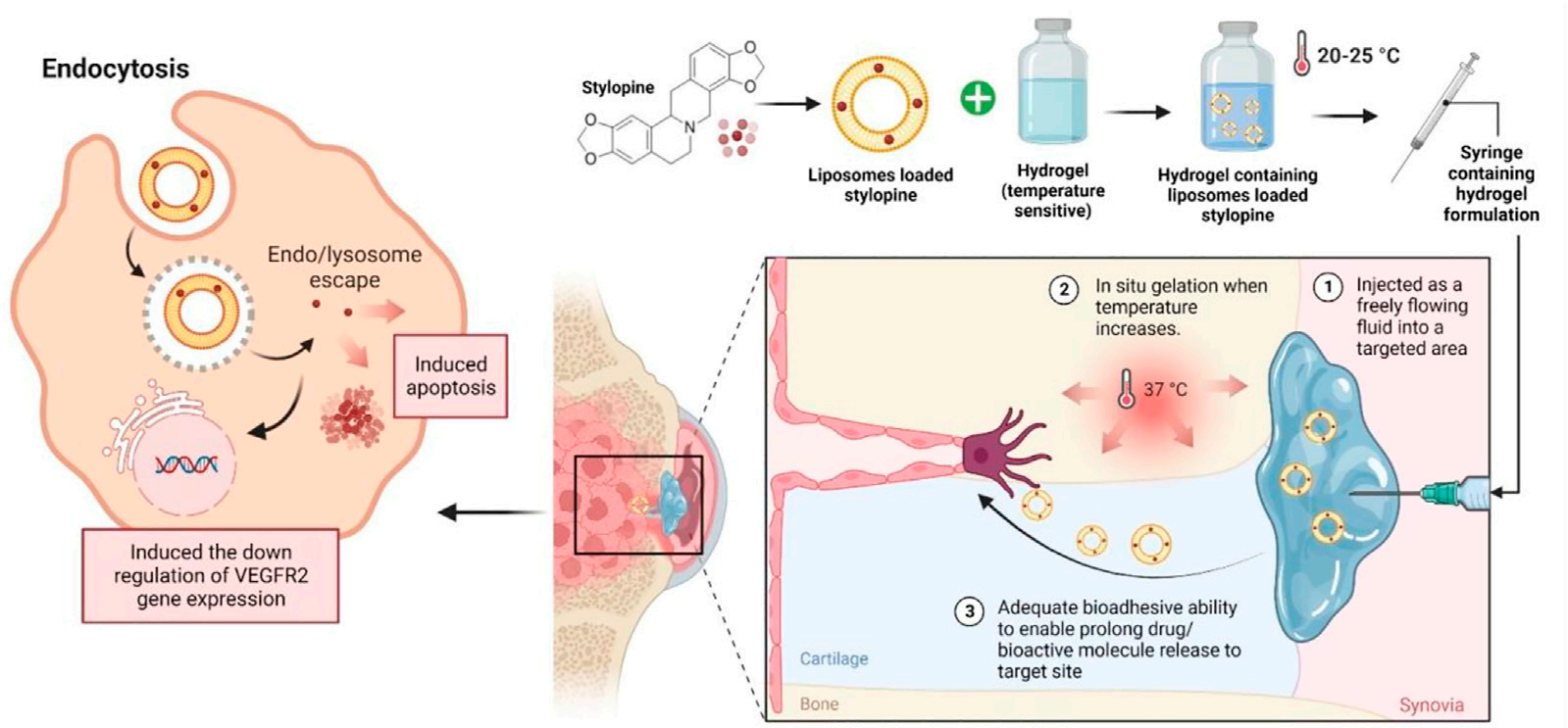
Future perspective of thermoresponsive hydrogel embedded liposomal stylopine for the management of osteosarcoma. Abbreviations: VEGFR2; Vascular endothelial growth factor receptor two.

Future research on optimising stylopine delivery can also be done to ensure optimal concentrations at the biological target site and decrease the frequency of high drug dosages, which could elevate the risk of systemic toxicity. The use of nanocarriers to load bioactive compounds has garnered the interest of researchers, particularly the usage of certain polymers to accomplish smart delivery, such as pH-sensitive (Fan et al., 2019; Andrade et al., 2021), thermoresponsive (Carreño et al., 2021; Ma and Yan, 2021; Rafael et al., 2021), and many more. The deposition of thermoresponsive hydrogels is thought to be one of the determining factors in enhancing the delivery and efficacy of any therapeutic agents, as earlier studies of such formulations have shown them to be effective against cancer. As a result, we proposed using injectable thermoresponsive hydrogels that can be deployed in liquid form and then changed to form a depot for the controlled release of stylopine to target osteosarcomas. Liposomes in the formulation, on the other hand, may increase the quantity of stylopine delivered directly into cancer cells by receptor-mediated endocytosis (Alshehri et al., 2018; Xiang et al., 2018), interfering with VEGFR2 protein expression as well as triggered apoptosis. Despite our expectation that the formulation will yield positive outcomes, extensive research into the behaviour of such formulations is required before progressing to clinical trials (Figure 13).

## Data Availability

The original contributions presented in the study are included in the article/supplementary materials, further inquiries can be directed to the corresponding authors.
