# Erratum: Stylopine: A potential natural metabolite to block vascular endothelial growth factor receptor 2 (VEGFR2) in osteosarcoma therapy

**DOI:** 10.3389/fphar.2023.1213155

**Published:** 2023-05-05

**Authors:** Frontiers Production Office

**Affiliations:** Frontiers Media SA, Lausanne, Switzerland

**Keywords:** benzylisoquinoline alkaloids, stylopine, MG-63, osteosarcoma, VEGFR2

Due to a production error, there was an error in affiliation 4. Instead of “Department of Foundation, Georgetown, Pulau Pinang, Malaysia,” it should be “Department of Foundation, RCSI & UCD Malaysia Campus, George Town, Pulau Pinang, Malaysia.”

The publisher apologizes for this mistake. The original version of this article has been updated.

