# Corrigendum: Stylopine: a potential natural metabolite to block vascular endothelial growth factor receptor 2 (VEGFR2) in osteosarcoma therapy

**DOI:** 10.3389/fphar.2024.1343756

**Published:** 2024-01-17

**Authors:** Naveen Kumar Velayutham, Tamilanban Thamaraikani, Shadma Wahab, Mohammad Khalid, Gobinath Ramachawolran, Shahabe Saquib Abullais, Ling Shing Wong, Mahendran Sekar, Siew Hua Gan, Angel Jemima Ebenezer, Mrinalini Ravikumar, Vetriselvan Subramaniyan, Nur Najihah Izzati Mat Rani, Yuan Seng Wu, Srikanth Jeyabalan

**Affiliations:** ^1^ Department of Pharmacology, SRM College of Pharmacy, SRM Institute of Science and Technology, Chennai, Tamil Nadu, India; ^2^ Department of Pharmacognosy, College of Pharmacy, King Khalid University, Abha, Saudi Arabia; ^3^ Department of Pharmacognosy, College of Pharmacy, Prince Sattam Bin Abdulaziz University, Al-Kharj, Saudi Arabia; ^4^ Department of Foundation, RCSI and UCD Malaysia Campus, George Town, Malaysia; ^5^ Department of Periodontics and Community Dental Sciences, College of Dentistry, King Khalid University, Abha, Saudi Arabia; ^6^ Faculty of Health and Life Sciences, INTI International University, Nilai, Malaysia; ^7^ School of Pharmacy, Monash University Malaysia, Subang Jaya, Selangor, Malaysia; ^8^ Center for Transdisciplinary Research, Department of Pharmacology, Saveetha Dental College, Saveetha Institute of Medical and Technical Science, Chennai, India; ^9^ Trichy Research Institute of Biotechnology Pvt Ltd., Trichy, Tamil Nadu, India; ^10^ Jeffrey Cheah School of Medicine and Health Sciences, Monash University Malaysia, Subang Jaya, Selangor, Malaysia; ^11^ Faculty of Pharmacy and Health Sciences, Royal College of Medicine Perak, Universiti Kuala Lumpur, Ipoh, Perak, Malaysia; ^12^ Department of Biological Sciences and Centre for Virus and Vaccine Research, School of Medical and Life Sciences, Sunway University, Subang Jaya, Selangor, Malaysia; ^13^ Department of Pharmacology, Sri Ramachandra Faculty of Pharmacy, Sri Ramachandra Institute of Higher Education and Research (DU), Chennai, Tamil Nadu, India

**Keywords:** benzylisoquinoline alkaloids, stylopine, MG-63, osteosarcoma, VEGFR2

In the published article, there was an error in the **Article Title**. Instead of “*Stylopine: A potential natural* metabolite to activate vascular endothelial growth factor receptor 2 (VEGFR2) in osteosarcoma therapy,” it should be “Stylopine: A potential natural metabolite to block vascular endothelial growth factor receptor 2 (VEGFR2) in osteosarcoma therapy.”

Additionally, there was an error in the **Conflict of Interest** statement. The original statement omitted the declaration for commercial affiliations. The correct **Conflict of Interest** statement appears below.

## Conflict of interest

Author AE was employed by Trichy Research Institute of Biotechnology Pvt Ltd.

The remaining authors declare that the research was conducted in the absence of any commercial or financial relationships that could be construed as a potential conflict of interest.

The authors apologize for this error and state that this does not change the scientific conclusions of the article in any way. The original article has been updated.

